# Enhancement of Antibacterial Activity of *Paludifilum halophilum* and Identification of N-(1-Carboxy-ethyl)-phthalamic Acid as the Main Bioactive Compound

**DOI:** 10.1155/2020/4805706

**Published:** 2020-02-12

**Authors:** Donyez Frikha-Dammak, Jawhar Fakhfakh, Dalel Belhaj, Emna Bouattour, Houda Ayadi, Moncef Chaabouni, Habib Ayadi, Sami Maalej

**Affiliations:** ^1^University of Sfax, Laboratory of Marine Biodiversity and Environment (LR18ES/30), BP 1171, CP 3000, Sfax, Tunisia; ^2^University of Sfax, Laboratory of Organic Chemistry (LR17ES/08) Natural Substances Section, BP 1171, CP 3000, Sfax, Tunisia; ^3^University of Sfax, Engineering Laboratory of Environment and Eco-Technology (LR16ES/19), BP 1173, CP 3038, Sfax, Tunisia

## Abstract

The aim of this study was to determine the combined effect of fermentation parameters and enhance the production of cellular biomass and antibacterial compounds from *Paludifilum halophilum* SMBg3 using the response surface methodology (RSM). Eight variables were screened to assess the effects of fermentation parameters on growth and metabolite production by Taguchi experimental design. Among these, the initial pH, temperature, and the percentage of MgSO_4_·7H_2_O in the medium were found to be most influential. The Box–Behnken design was applied to derive a statistical model for the optimization of these three fermentation parameters. The optimal parameters were initial pH: 8.3, temperature growth: 44°C, and MgSO_4_·7H_2_O: 1.6%, respectively. The maximum yield of biomass and metabolite production were, respectively, 11 mg/mL dry weight and 15.5 mm inhibition zone diameter against *Salmonella enterica*, which were in agreement with predicted values. The bioactive compounds were separated by the thick-layer chromatography technique and analyzed by GC/MS, NMR (1D and 2D), and Fourier-transform infrared spectroscopy (FT-IR). In addition to several fatty acids, N-(1-carboxy-ethyl)-phthalamic acid was identified as the main antibacterial compound. This element exhibited a potent activity against the ciprofloxacin-resistant *Salmonella enterica* CIP 8039 and *Pseudomonas aeruginosa* ATCC 9027 with a minimum inhibitory concentration (MIC) value range of 12.5–25 *μ*g/mL. Results demonstrated that *P. halophilum* strain SMBg3 is a promising resource for novel antibacterial production due to its high-level yield potential and the capacity for large-scale fermentation.

## 1. Introduction

The increasing request for novel antibiotics is chiefly ascribed to the emergence of several multidrug-resistant pathogens around the globe, along with the drastically shrinking number of new potentially detected molecules [1, 2]. In this respect, *Actinobacteria* still figure on the list of the most promising biological sources of new natural products due to its unique adaptation characteristics [3, 4]. However, the intensive exploration of terrestrial actinomycetes along the1950–70s has led to frequent rediscoveries of the same bioactive compounds. In contrast, the unexplored and underexplored marine environments, mainly extreme ones, are promising sources of rare actinomycetes believed to be rich sources of new interesting compounds [4, 5].

Examples of extreme marine niches include natural (seas, marine stromatolites, and salt marshes) and artificial (salterns and crystallizer ponds) hypersaline habitats. These environments, endowed with high salinity, nutrient deficiency, and strong oxidative stress, were shown to harbor several species of halotolerant and halophilic actinomycetes, ranging from typical representatives of *Streptomyces* to more exotic and rare *Salinispora, Marinophilus, Salinibacterium, Aeromicrobium, Nocardiopsis* and *Actinopolyspora* species [6, 7]. On the basis of phenotypic, chemotaxonomic, and phylogenetic classification, several genera of these actinomycetes such as *Mechercharimyces*, *Melghirimyces, Croceifilum*, and *Salinithrix* were affiliated to the *Thermoactinomycetaceae* family of the phylum *Firmicutes* [8–10]. This increasing number of nonconventional actinomycetes considerably paves the way for the discovery of new bioactive natural products [11, 12].

The discovery of novel strains of halophilic actinobacteria seems to be the first and the key step toward obtaining novel bioactive compounds and subsequently to design natural product-based drugs. For instance, lajollamycin was isolated from the new strain *Streptomyces nodosus* [6], while salinosporamide A and borrelidins C–E were obtained from *Salinispora tropica* [13] and *Nocardiopsis* sp. [14], respectively. These compounds are now a special target of several biotechnological industrial and academic institutions, since they possess a strong potential to be developed into pharmaceutical products [15]. The second significant step that influences the detection of novel drugs could be the enhancement of the bioactive compound's production level to a threshold detectable by the methods of analysis, which could be attained by the optimization of the composition of the fermentation medium and the culture conditions [16]. Indeed, the source and concentration of nutrients along with the cultural conditions are known to have mysterious effects on bioactive metabolite production in actinomycetes [17–19]. Minor changes in the nature of medium composition and condition have been reported to affect antibiotic biosynthesis. Thus, designing a suitable medium and convenient condition for cultivation has prime significance in improving the antibiotic yield. The approach based on single parameter per trial used in conventional media optimization technique is an arduous and time-consuming process, involving a large number of experimental trials to determine the optimum levels of all variables. Besides, it may not provide the combined effect between different parameters and frequently fails to identify the variables that provide an optimum response. As a practical alternative, it is of great interest to develop a technique which involves statistical optimization to undermine those issues related to conventional optimization. Response surface methodology (RSM), a compilation of mathematical and statistical techniques for building empirical models, has been recognized as an attractive approach in terms of improving the production of commercially important bioactive metabolites. This approach has been successfully applied in secondary metabolites production [20, 21] and particularly in enhanced antibiotic production by actinobacteria [22–25].

In a previous study, we performed a screening procedure to isolate the novel Thermoactinomycete *Paludifilum halophilum* DSM 102817^T^ from the superficial sediment of the solar saltern of Sfax, Tunisia, which was assigned to a new genus of the family of *Thermoactinomycetaceae* [26]. In the present study, RSM based on Box–Behnken design was used to optimize the components of Bennett's medium for maximum biomass and antibacterial metabolite production. Moreover, via GC/MS, 1D and 2D NMR, and FT-IR spectroscopic analyses, the N-(1-carboxy-ethyl)-phthalamic acid was reported as the main bioactive compound.

## 2. Materials and Methods

### 2.1. Microorganisms

*Paludifilum halophilum* strain SMBg3 was kept in 20% (v/v) glycerol at −80°C. The working strain was prepared on Bennett's medium agar, supplemented with 10% NaCl, and stored at 4°C. For antibacterial assay, six cultures labelled *Salmonella enterica* CIP 8039, *Escherichia coli* ATCC 8739, *Pseudomonas aeruginosa* ATCC 9027*, Staphylococcus aureus* ATCC 6538, *Listeria ivanovii* BUG 496, and *Micrococcus luteus* LB141110 were used.

### 2.2. Basal Medium Screening for Antibacterial Activity

Strain SMBg3 was initially cultivated in 50 mL of Bennett's medium supplemented with 10% NaCl. After 3 days of growth on a rotary shaker at 37°C and at 200 rpm, 5 mL of the preculture was inoculated in 50 mL of five different basal media labelled Bennett's (Bennett's), Glucose Yeast Peptone (GYP), Glucose Yeast Malt (GYM), NPB (NPB), and Glycerol Starch (GSA). After 7 days of growth at similar conditions, crude metabolites were extracted from culture broths as described in Frikha-Dammak et al. [27]. After the removal of mycelia biomass from culture broth by filtration through a tared filter (Sartorius; cellulose nitrate; pore size, 0.2 *μ*m) and the determination of the dry weight at 105°C, the entire supernatant was extracted twice with ethyl acetate. The ethyl acetate was concentrated in vacuum to dry material, redissolved in 1 mL of ethyl acetate, and then assessed in triplicate for antibacterial activity against *Salmonella enterica* by the disc diffusion method. Test culture was plated from the LB medium on Mueller-Hinton agar and then dried for 30 min before applying 6 mm sterile discs loading with 10 *μ*L of each extract. A sterile disc impregnated with ethyl acetate was used as control. After being stored at 4°C for 2 h, the plates were incubated at 37°C for 18–20 h. The bioactivity of extracts was evaluated by measuring manually the inhibition zone (IZ) around the disc with an accuracy of  ± 1 mm.

### 2.3. Optimization of Biomass and Antibacterial Activity Using Box–Behnken Design

In order to improve biomass and antibacterial metabolites production by Bennett's medium, a Taguchi experimental design with eight variables including various carbon and nitrogen sources, inorganic salts, and physicochemical parameters was used to select the most influential factors. The contribution of the individual parameter was determined by analysis of variance (ANOVA). Temperature of the culture (*X*_1_), pH of the medium (*X*_2_), and the percentage of MgSO_4_·7H_2_O added to Bennett's medium (*X*_3_) were selected as the most influential parameters and optimized through conventional means. To determine the combined effect of the three selected parameters, Box–Behnken design, using NemrodW software with 19 runs, was applied [28]. These three independent variables were carried out at three levels: low, middle, and high coded as −1, 0, and +1, respectively, simultaneously with four replicates of the central points. The regression analysis was performed to estimate the response function as a second-order polynomial equation:

(1) $$\begin{array}{c} Y=\;b_{0}+b_{1}\times X_{1}+b_{2}\times X_{2}+b_{3}\times X_{3}+b_{11}\times \left(X_{1}\times X_{2}\right)+b_{22}\times \left(X_{2}\times X_{2}\right)+b_{33}\times \left(X_{3}\times X_{3}\right)+b_{12}\times \left(X_{1}\times X_{2}\right)+b_{13}\times \left(X_{1}\times X_{3}\right)+b_{23}\times \left(X_{2}\times X_{3}\right), \end{array}$$

where *Y* denotes the predicted response, *X*_1_, *X*_2_, and *X*_3_ denote the independent variables, *b*_0_ is the model constant, *b*_1_, *b*_2_, and *b*_3_ are the linear effects, *b*_11_, *b*_22_, and *b*_33_ are the squared effects, and *b*_12_, *b*_13_, and *b*_23_ are the interaction effects between two factors. The statistical software NemrodW was applied for the regression analysis of the experimental data, the quadratic model building as well as plotting two-dimensional isoresponse curves and three-dimensional response surface graphs. This measure aimed at establishing the relationship between the responses and the experimental levels of each independent variable. To attest the good quality of the fitting, we calculated the values of the coefficient of determination (*R*^2^). The fitted polynomial equation was then validated through an analysis of variance (ANOVA) and exploited to search for optimal conditions.

### 2.4. Separation of Antibacterial Products and Structural Determination

After 7 days of incubation at 44°C, 10 L of optimized Bennett's medium was combined and mycelia of *P. halophilum* were discarded by filtration. The mycelium-free medium supernatant was extracted with an equal volume of ethyl acetate, evaporated, and resuspended in ethyl acetate with a stock solution of 20 mg/mL. The TLC technique was used to separate the components of the concentrated crude extract. For this, a 2 *μ*L aliquot of the stock solution was spotted onto the silica gel plate at a position 1.5 cm above the bottom of the plate. Thirty milliliters of 5% methanol in chloroform was added to the developing chamber, at a depth of 0.5 cm. The spotted TLC plate was placed into the developing chamber and sealed with a glass lid. Separation was concluded when the development solvent front reached a position at 1 cm from the top of the TLC plate. The plate was then transferred to an oven and allowed to dry at 80°C for 5 min. The separated “spots” were observed on the plate under UV light at 254 nm (absorbance) and 365 nm (fluorescence), scraped from the plate in fresh mobile phase, filtered, and screened for antibacterial activity against *S. enterica* as described above. Products from the most active F2 fraction were characterized through GC/MS, NMR, and FT-IR analyses.

A GC/MS analysis (Agilent Technologies, 5975 B Inert MSD Mass-Selective Detector, France) was performed with a HP-5MS (phenyl-methyl siloxane) column (30 m × 250 m × 0.25 m). High-purity helium was used as the carrier gas at a flow rate of 1 mL/min. The temperatures of the injector and detector were 250 and 240°C, respectively. The GC column temperature was programmed from 120°C (initial equilibrium time 5 min) to 180°C *via* a ramp of 3°C/min and 180–220°C *via* a ramp of 10°C/min and maintained at 220°C for 31 min. The MS operates with electron impact ionization in full-scan mode from 30 *m*/*z* to 700. The inlet and MS transfer line temperatures were maintained at 250°C, and the ion source temperature was 200°C. Sample injection (1 *μ*L) was in splitless mode.

^1^H and^13^C NMR spectra of the fraction F2 were acquired at 400 and 100 MHz, respectively, on a Bruker Avance AMX 400 NMR. The spectra were recorded at 25°C for 20 mg of the dissolved fraction in 1 mL of CDCl_3_. The chemical shifts for ^1^H and ^13^C were relative to those of residual solvent. All the ^1^H and ^13^C signals were assigned on the basis of the chemical shifts, spin-spin coupling constants, splitting patterns, and signal intensities, as well as by means of ^1^H-^1^H COSY, ^1^H-^13^C HSQC, and ^1^H-^13^C HMBC experiments.

The infrared spectrum of the same fraction was recorded on Shimadzu IR-470 model. The spectrum was scanned in the range of 400 to 4,000 cm^−1^ using a potassium bromide pellet and plotted as intensity versus wavelength.

### 2.5. Antibacterial Evaluation of TLC Separated F2 Fraction

The TLC separated F2 fraction was screened against a panel of Gram-positive and Gram-negative type culture bacteria, as indicated in Table 1. MIC assays were performed in duplicate in 96-well microtiter plates on the basis of the protocol recommended by the Clinical and Laboratory Standards Institute [29]. Stock solutions of F2 fraction in 1 mg dimethyl sulfoxide (DMSO)/mL were added to the first well in a row and serially diluted (twofold per transfer) across the microtiter plate. The DMSO was used as a negative control. Overnight cultures of bacteria in LB medium were diluted 1,000-fold, and 10 *μ*L was used as an inoculum in each well. MIC values were determined after 24 h incubation (37°C, static growth) and 3-(4, 5-dimethylthiazol-2-yl)-2,5-diphenyl-tetrazolium bromide (MTT) (Sigma-Aldrich) was used for growth visualization. The MIC was defined as the lowest concentration, devoid of a color variation from yellow to purple.

### 2.6. Statistical Analysis

All data regarding the antibacterial activity were repeated three times, subjected to statistical analysis using SPSS software package (Windows version 20.0; IBM SPSS) and expressed as mean ± standard deviation (SD). Graphs were schemed using the computer program Origin Pro 8.5.1 (Origin Lab, Corporation, Northampton, USA).

## 3. Results

### 3.1. General Properties of *P. halophilum*

Strain SMBg3 was isolated from a surface sediment sample collected from the solar saltern of Sfax, Tunisia. The sediment suspension was diluted in saline water (150 g/L) and spread over the surface of *Streptomyces* isolation agar medium. On this medium, the isolate had a pale-yellow-colored aerial mycelium, with long chains containing fluorescent and circular spores. Colonies, 4.0–8.0 mm in diameter, were circular and irregular with wrinkles between the center and the edge of the colony. Phenotypic characteristics of the isolate SMBg3 such as the ability to tolerate NaCl up to a concentration of 20% (w/v) and the absence of growth at salinities lower than 5% were not typical for halophilic actinomycete strains, prompting phylogenetic analysis of its 16S rRNA gene.

A dendrogram based on the almost complete 16S rRNA gene (1401 pb) clearly showed that SMBg3 belongs to an independent phylogenetic lineage of the family of *Thermoactinomycetaceae*, strongly suggesting that this strain represents a novel species of a new genus of this family for which the name *Paludifilum halophilum* is proposed and the type strain of the type species is SMBg3^T^ (=DSM 102817^T^ = CCUG 68698^T^) [26].

### 3.2. Selection of Basal Medium and Determination of Most Influential Parameters

**Figure 1:**
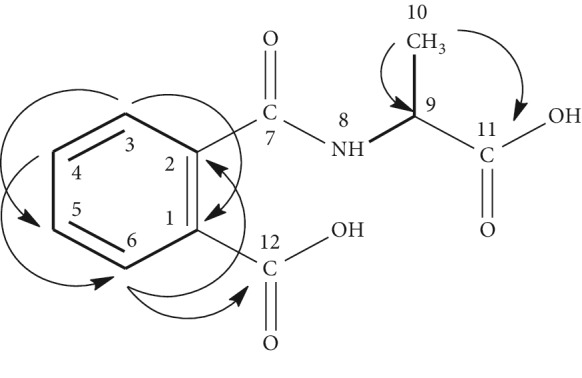
Effects of different temperatures (a), pH (b), and % of MgSO4·7H2O (c), on biomass production (–) and crude extract antibacterial activity (◆) of *P. halophilum* against *S. enterica*.

In order to select a preliminary basal medium for growth and antibacterial metabolites production, *P. halophilum* crude extracts from 5 different 7-day culture broth were compared for their antibacterial activity against *S. enterica*. Based on results from [Supplementary-material supplementary-material-1], Bennett's medium was selected for further optimization. A Taguchi experimental design with eight variables and 18 runs was used to select the most influential factors on growth and antibacterial activity (data not shown). Temperature, pH, and % MgSO_4_·7H_2_O were selected and optimized through conventional means based on the single factor variation (Figure 1). The effect of temperature variation was assessed in Bennett's medium, while pH and MgSO_4_·7H_2_O rated were fixed at 7.4 and 0%, respectively. As shown in Figure 1(a), the maximum biomass and antibacterial activity were observed at 40°C, while slight decreases were obtained at 37 and 45°C. Therefore, the temperature range of 37–45°C was considered optimal in the optimization experiments. The effect of pH was also investigated at different values between 5 and 10, while temperature culture and MgSO_4_·7H_2_O were fixed, respectively, at 40°C and zero percent. As shown in Figure 1(b), biomass and antibacterial activity increased and reached maximum levels at pH 8. At pH 7.4 and 9, both biomass and bacterial activity exhibited significant decreases. This range of pH was deemed optimal in the experiments.  Figure 1(c) displayed the effect of the percentage of MgSO_4_·7H_2_O on growth and antibacterial activity, when temperature and pH were fixed at 40°C and 8, respectively. A slight increase in both parameters was observed at 1% MgSO_4_·7H_2_O. However, at higher concentrations, a slight decrease of the biomass was observed, while antibacterial activity was maintained almost constant. Thus, the MgSO_4_·7H_2_O percentage range between 1 and 2% was chosen as optimal for further optimization experiments.

### 3.3. RSM for the Optimization of the Influential Parameters

Optimization of biomass production and antibacterial activity of *P. halophilum* SMBg3 was continued by determining the optimal concentration of the combined three influential parameters (*T*, pH, and MgSO_4_·7H_2_O) by means of the RSM methodology and the central composite design (CCD). The respective low and high levels of the three parameters are summarized in Table 2. A set of 19 experiments (including three checkpoints) with different level combinations, chosen according to a three-variable Box–Behnken design, was carried out, and the observed and predicted results for biomass production and antibacterial activities after 7 days of culture are presented in Table 3. The best growth with a final dry biomass concentration of 11 mg/mL and the best antibacterial activity with an inhibition zone of the growth of *S. enterica* of 15.5 mm were obtained in run 8. The experimental responses were analyzed by the NemrodW software, and the coefficients of the postulated model (equation (1)) were calculated by the least-squares method. The fitted models Y1 and Y_2_ expressed in coded variables were represented by the following equations:

(1) $$\begin{array}{c} Y_{1}=15.77+0.74X_{1}+0.38X_{2}+0.08X_{3}+3.5X_{1}^{2}-0.91X_{2}^{2}-0.79X_{3}^{2}-0.89X_{1}X_{2}+0.37X_{1}X_{3}+0.42X_{2}X_{3},\\ Y_{2}=11.47-0.20X_{1}-0.30X_{2}+0.64X_{3}+0.36X_{1}^{2}+0.20X_{2}^{2}+1.02X_{3}^{2}+0.99X_{1}X_{2}+1.63X_{1}X_{3}+0.31X_{2}X_{3}, \end{array}$$

where *Y*_1_ and *Y*_2_ represent the biomass production and antibacterial activity against *S. enterica*, respectively, and *X*_1_, *X*_2_, and *X*_3_ are the coded factors of temperature, pH, and percentage of MgSO_4_·7H_2_O, respectively.

To attest the good quality of the fitting, an analysis of variance was carried out as shown in Table 4. Results indicated that the regression sum of squares is statistically significant (*p* < 0.05). The convenience of the model developed for two responses was also evaluated by the coefficient of determination (*R*^2^), which was found to be 0.8. This means that 80% of the observed variations are attributed to the variable effects, indicating that the observed response values were close to those of predicted responses calculated from the model. On the other hand, the validity of the model was established by comparing the variance related to the lack of fit (lof) to that of pure error, which demonstrated the nonsignificance of the lof (Table 4). The adequacy of the predicted model was further verified by three additional independent experiments carried out at three checkpoints. The measured responses were in close agreement with the calculated values, as illustrated in [Supplementary-material supplementary-material-1].

**Figure 2:**
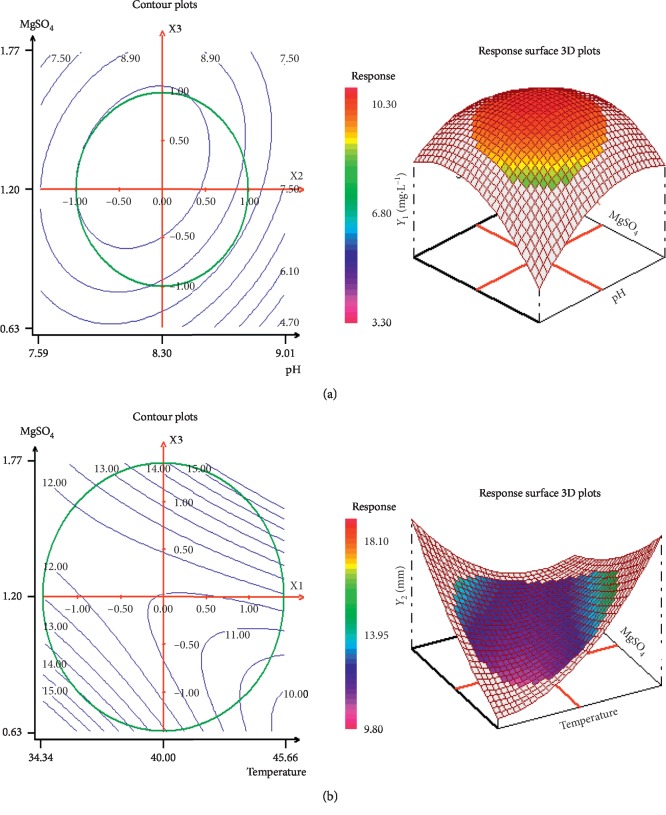
Response surface contour and 3D plots showing interactive effects of pH and MgSO_4_·7H_2_O when *X*_1_ = 1 on biomass production (a) and effects of temperature and MgSO_4_·7H_2_O when *X*_2_ = 1 on antibacterial activity (b).

Results of the combined effect of the selected parameters (*T*, pH, and % of MgSO_4_·7H_2_O) on the growth and antibacterial activity of *P. halophilum* were shown in the form of two-dimensional (2D) and three-dimensional (3D) plots (Figure 2). Each 3D NemrodW plot presented the effects of two variables, while the remaining one was held at the middle level. Results from Figures 2(a) and 2(b) revealed that the pH and % of MgSO_4_·7H_2_O have a significant effect and they increase the biomass production, whereas temperature and % of MgSO_4_·7H_2_O have significant positive effects on antibacterial activity (Figure 2(b)). Based on the NemrodW software results, the optimum conditions for maximizing biomass and antibacterial metabolites production could be predicted as follows: 44°C for incubation temperature, medium pH 8.3, and 1.6% MgSO_4_·7H_2_O supplementation. The validation of the statistical results using the optimized medium was accomplished by carrying out shake-flask experiments in triplicate. The maximum biomass of 11 mg/mL and antibiotic activity of 15.5 mm obtained experimentally were found to be in close agreement with the predicted values of 9.67 mg/mL and 14.92 mm, respectively. Therefore, the developed model was considered to be accurate and reliable for predicting the production of biomass and antibacterial activity by *P. halophilum* SMBg3. The final optimized medium contained glucose (10 g), meat extract (1 g), yeast extract (1 g), peptone (2 g), NaCl (100 g), and MgSO_4_·7H_2_O (16 g) in 1 L of distilled water adjusted to pH 8.3 and incubated at 44°C. Under these conditions, the biomass concentration and antibacterial activity against *S. enterica* after 7 days of fermentation were improved by 83% and 63%, respectively, compared to nonoptimized conditions.

### 3.4. Characterization of Antibacterial Compounds from F2 Fraction

Among the three fractions (F1, F2, and F3) obtained by thick-layer chromatography, the F2 fraction having an Rf value of 0.87 had the most antibacterial activity against *S. enterica* (data not shown). After being scraped from the TLC plate, dissolved in chloroform, filtered, and evaporated, this fraction yielded 23 mg of a white powder. In an attempt to identify the chemical structure of its bioactive compounds, this fraction was firstly subjected to GC/MS analysis. The obtained results revealed the presence of several fatty acids along with other peaks that they could not be identified by this technique ([Supplementary-material supplementary-material-1]). Therefore, in the aim to identify the unknown compounds, F2 was subjected to NMR analysis. The ^1^H NMR spectrum ([Supplementary-material supplementary-material-1]) distinguished intense signals related, mainly, to saturated fatty acids. From the H-H COSY ([Supplementary-material supplementary-material-1]), spectrum connections were depicted between the multiplet at 0.86 ppm (CH_3_) and the intense signal at 1.3 ppm (CH_2_)_*n*_ which correlates with the multiple at 1.6 ppm due to the methylene groups in *β* position to the carboxyl groups. The latter correlated, in turn, with the methylene groups at 2.34 ppm of the position to the carboxyl groups. The related resonances on the ^13^C NMR spectrum ([Supplementary-material supplementary-material-1]) raised at 13.98 ppm (CH_3_), 24.7–24.9 ppm (CH_2_-CH_2_-COO), 34.1 ppm (CH_2_-CH_2_-COO), and 175.1 ppm due to the carbonyl groups of the acid moieties. The study of the HSQC spectrum, together with the HMBC spectrum ([Supplementary-material supplementary-material-1] b, c), corroborates the global fatty acids' skeletons, as detailed in [Supplementary-material supplementary-material-1], whose signals cannot be distinguished in the ^1^H and ^13^C spectra due to their closer structure.

On the other hand, the ^1^H NMR spectrum exhibited in its aromatic region an obvious spin system of an *ortho* substituting aromatic ring by the doublet at 8.30 ppm (*J* = 8.2 Hz) and correlating with a triplet at 7.47 ppm (*J* = 8.4 Hz) which correlates, in turn, with another triplet at 7.14 ppm (*J* = 7.5 Hz) that makes another correlation with a doublet at 7.55 ppm (*J* = 7.8 Hz) in the H-H COSY experiment ([Supplementary-material supplementary-material-1]). The latter displayed other correlations between a doublet at 6.94 ppm (N-H), which exhibited no correlation in the HSQC spectrum ([Supplementary-material supplementary-material-1]) and the multiplet at 4.98 ppm related to a methyl's proton. This latter makes correlation with the doublet of three protons at 1.60 ppm, related to a methyl group. HMBC spectrum ([Supplementary-material supplementary-material-1]) exhibited connectivity between the multiplet at 4.98 ppm (C-H) and each of the peaks at 170.5 ppm related to the carbonyl of an amid group, at 19.1 ppm due to the methyl group and at 170.5 ppm due to the carbonyl group of a carboxyl moiety. In addition, this spectrum displayed correlations between the doublet at 7.55 ppm and the peak at 167.9 ppm, relating to the carbonyl of a carboxyl moiety. All of the above data permitted to identify this compound as N-(1-carboxy-ethyl)-phthalamic acid and to assign all of its ^1^H and ^13^C chemical shifts (Table 5).

**Figure 3:**
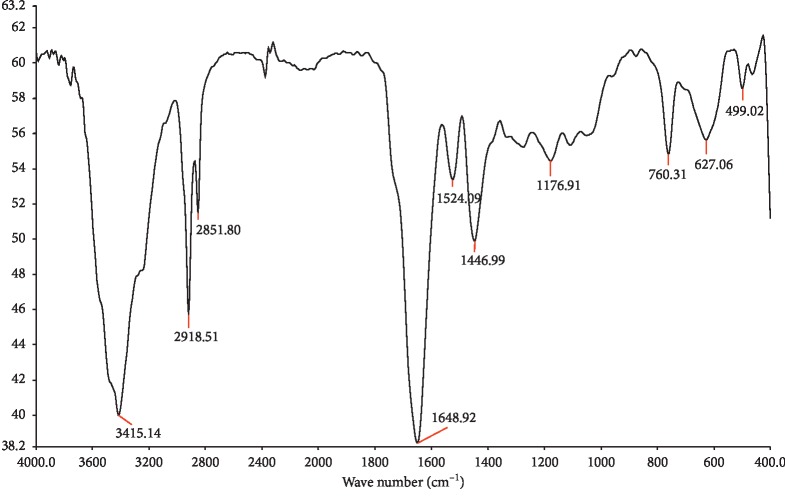
FT-IR analysis of F2 fraction ethyl acetate extract.

The FT-IR spectrum (Figure 3) supplies information that consolidates the presence of the identified compound in F2 by showing a large absorbance band between 3000 and 3600 cm^−1^ related to O-H and N-H stretching vibrations, two sharp bands at 2918 and 2851 cm^−1^ due to the CH stretching vibration of aliphatic CH, CH_2_, and CH_3_ groups, a broad and intense band centered at about 1648 cm^−1^ related to stretching vibrations of the amid and acid carbonyl groups, a medium intense band at 1446 cm^−1^ corresponding to CH_2_ and CH_3_ bending vibrations, and three weak bands at 1269–1176–1102 cm^−1^ due to C–O and C–C stretching vibrations. The aromatic C=C which usually yields absorption bands at 3000 cm^−1^ could be overlapped in this case with the large band relating to O-H and N-H vibrations.

The partial characterized N-(1-carboxy-ethyl)-phthalamic acid was tested against a panel of Gram-negative and Gram-positive pathogens (Table 1). Among Gram-negative bacteria, potent activity was observed against the ciprofloxacin-resistant *Salmonella enterica* and against *Pseudomonas aeruginosa* with a range of MIC values between 12.5 and 25 *μ*g/mL. However, among Gram-positive bacteria, only *Staphylococcus aureus* exhibited moderate susceptibility to the compound with MIC values of 25–50 *μ*g/mL. The specific inhibitory activity against *S. enterica* indicated that the N-(1-carboxy-ethyl)-phthalamic acid, approximately as potent as the positive control ampicillin, might be of particular interest for treating different cases of salmonellosis.

## 4. Discussion

Salterns are the most extreme habitats in terms of salinity and they can harbor truly halophilic actinomycetes. However, the chemistry of actinobacteria in these hypersaline environments had been mostly neglected until the report of Kim et al. [30], on the production of saltern amides with the ability to induce apoptosis in human cancer cells, as the first secondary metabolites from a saltern-derived *Streptomyces* sp. So far, several bioactive compounds such as xiamycins C–E from a halophilic *Streptomyces* sp., with potent antiviral activity against porcine epidemic diarrhea virus (PEDV) [12] and borrelidins C–E from a saltern-derived halophilic *Nocardiopsis* sp. with antibacterial activity against *S. enteric* [14], have been discovered from halophilic actinomycetes. In a relentless effort to investigate the promising biosynthetic potential of cultivable saltern-derived actinomycete, the present study was conducted to improve the antibacterial activity of the saltern-based *Paludifilum halophilum* SMBg3 by Box–Behnken design and to carry out the extraction, identification, and biological evaluation of the main active compounds.

In view of significance of media, we attempted to select a basal medium for the growth of *Paludifilum halophilum* and optimize their components and culture conditions for enhanced biomass production and antibacterial activity. By Taguchi design and conventional means, we revealed that the MgSO_4_·7H_2_O, supplemented to the selected basal Bennett's medium, pH, and temperature of growth significantly affected the biomass and antibacterial compound production by *P. halophilum* SMBg3. Positive effect of MgSO_4_·7H_2_O on antibiotic production by actinomycetes has previously been reported by several researchers. For instance, Zhu et al. [31] reported that the production of avilamycin in *Streptomyces viridochromogenes* was significantly increased in the medium supplemented with MgSO_4_·7H_2_O. The findings of Yang et al. [32], pertaining to *Xenorhabdus sp* strain D43, and Wang et al. [33], related to the actinomycete *Saccharothrix yanglingensis* strain Hhs.015T, also supported the importance of pH, *T*, and MgSO_4_·7H_2_O on the enhancement of the antibacterial metabolite production. To optimize the levels of influencing parameters, RMS with Box–Behnken design often proved to be a powerful tool. As an illustration, Wang et al. [16], applied this approach to medium optimization for antibiotic production by *Xenorhabdus bovienii* and recorded 37.8% increase in antibiotic activity. Recently, Rajeswari et al. [34] have reported a 78.8% increase in antibacterial activity by the saltern-based *Streptomyces sp*. strain JAJ13. In our study on *P. halophilum*, this approach allowed the determination of optimum levels of pH, *T*, and MgSO_4_·7H_2_O that favored 83% increase in biomass and 63% in antibacterial activity. The goodness of fit of the model was checked by using the coefficient of determination (*R*^2^), which provides a measure of variability in the observed response, explained by the experimental factors and their interactions [18]. The obtained value of 0.8% indicated that 80% of total variations could be explained by the model.

From the results of TLC analysis of the ethyl acetate crude extract of the *P. halophilum* culture, three fractions were obtained and the most active one against the ciprofloxacin-resistant *Salmonella enterica* CIP 8039 was fraction F2, which had a significant inhibition zone of 15 mm by disk diffusion assay compared to only 8 mm of ciprofloxacin on the same strain and at the same concentration of 30 *μ*g/disk. Based on 1D and 2D NMR and FT-IR data, in addition to several minor fatty acids, the main compound identified in the F2 fraction was N-(1-carboxy-ethyl)-phthalamic acid. To the best of our knowledge, there is no previous report of antibacterial activities associated with this compound and only several synthetic derivatives, such as phthalimide, showed to have analgesic activity and antiviral properties [35]. Orzeszko et al. [36] revealed that the incorporation of L-alanine and L-phenylalanine into the phthalimide allows the best antibacterial synthetic drug. Ivanova et al. [37] pointed out that phthalic acid diethyl ester, isolated from *Streptomyces* strain 1010, exerts an antibacterial activity against *Micrococcus luteus* (MIC, 3 *μ*g/ml); *Bacillus subtilis* (MIC, 12 *μ*g/mL), and *Staphylococcus aureus* (MIC, 25 *μ*g/mL). Such a N-(1-carboxy-ethyl) insertion on phthalamic acid is encountered for the first time in secondary metabolites from a microorganism and specifically from an actinomycete. Interestingly, the partially characterized N-(1-carboxy-ethyl)-phthalamic acid from *P. halophilum* has a strong inhibitory activity against ciprofloxacin-resistant *Salmonella enterica* and against *Pseudomonas aeruginosa* with an activity that equals the ampicillin positive control. Based on its spectrum of activity on ciprofloxacin-resistant bacteria, it could be suggested that N-(1-carboxy-ethyl)-phthalamic acid has bacterial DNA gyrase as a target site [38].

Finally, this study proves that the cultivable saltern-based actinomycete *P. halophilum* could be a potential candidate for novel antibacterial compounds and it deserves to be explored in depth. The identification of N-(1-carboxy-ethyl)-phthalamic acid as the first secondary metabolite from this strain emphasizes the unexplored potential of several halophilic rare actinomycetes, inhabiting extremely saline environments. Further studies are underway in our laboratory to improve the purification of the product and explore its cellular target and its structure-activity relation.

## 5. Conclusion

The halophilic bacterium *Paludifilum halophilum* strain SMBg3, isolated from a coastal solar saltern in Sfax (Tunisia), was shown to produce N-(1-carboxy-ethyl)-phthalamic acid, a new antibacterial compound, along with several fatty acids. The structure of N-(1-carboxy-ethyl)-phthalamic acid was determined by one- and two-dimensional NMR and FT-IR spectroscopy. N-(1-carboxy-ethyl)-phthalamic acid inhibited the growth of the ciprofloxacin-resistant *Salmonella enterica* CIP 8039 and *Pseudomonas aeruginosa* with low MIC values of 12.5–25 *μ*g/mL. On the basis of literature data and this work, derivatives of phthalamic acid such as N-(1-carboxy-ethyl)-phthalamic acid can be seen as a starting point to define a new, easily accessible scaffold in the search for new antibiotic agents against fluoroquinolone-resistant pathogens.

## Figures and Tables

**Table 1:** Activity spectrum of N-(1-carboxy-ethyl)-phthalamic acid and the positive control ampicillin against 6 types of cultures. The values in the table are representative of the range of MICs (*μ*g/mL) determined in three independent experiments.

Organism(acquired resistance)	N-(1-carboxy-ethyl)-phthalamic acid	Ampicillin
Gram-negative bacteria		
*Salmonella enterica* CIP	12.5–25	25
*Escherichia coli* ATCC 8739 (E, VA)	>200	12.5
*Pseudomonas aeruginosa* ATCC 9027	12.5–25	12.5

Gram-positive bacteria		
*Staphylococcus aureus* ATCC 6538	25–50	25
*Listeria ivanovii* BUG 496	>100	25
*Micrococcus luteus* LB 14110	>100	25

**Table 2:** Independent variables and their levels for Box–Behnken design.

Factor	Independent variables	Coded levels		
		−1	0	+1
*X *_1_	Temperature (°C)	36	40	44
*X *_2_	pH	7.8	8.3	8.8
*X *_3_	% MgSO_4_·7H_2_O (w/v)	0.8	1.2	1.6

**Table 3:** Box–Behnken design with observed and predicted values of biomass production (*Y*_1_) and antibacterial activity (*Y*_2_) after 7 days of *P. halophilum* SMBg3 culture.

Run	Independent variable			*Y *_1_: biomass production (mg/mL)		*Y *_2_: antibacterial activity (mm)	
	*X *_1_ (*T*, °C)	*X *_2_ (pH)	*X *_3_ (% MgSO_4_·7H_2_O)	Observed	Predicted	Observed	Predicted
1	−1	−1	0	7	6.34	12	11.55
2	1	1	0	8.30	9.6	13	13.13
3	−1	1	0	10.2	8.88	13	12.92
4	1	1	0	7.80	8.57	10	10.54
5	−1	0	−1	6.6	8.03	13.5	14.04
6	1	0	−1	9.6	8.76	10.5	10.37
7	−1	0	1	6.6	7.45	12	12.06
8	1	0	1	11	9.67	15.5	14.92
9	0	−1	−1	4.7	4.02	12.7	12.66
10	0	1	−1	3.7	3.94	11.7	11.43
11	0	−1	1	3.3	3.52	13	13.33
12	0	1	1	4.1	4.94	13.3	13.33
13	0	0	0	6.6	5.76	10	11.47
14	0	0	0	5.6	5.76	11.50	11.47
15	0	0	0	4.2	5.76	13	11.47
16	0	0	0	4.6	5.76	11	11.47

**Table 4:** Analysis of variance (ANOVA) for the selected quadratic models of biomass production (*Y*_1_) and antibacterial activity (*Y*_2_).

Source of variation	Sum of square	Degree of freedom	Mean square	Ratio	Significance
*Y *_1_					
Regression	67.5495	9	7.5	3.73	0.0315^*∗*^
Residual	18.1084	9	2.01		
Validity	14.6384	6	2.43	2.1	0.288 ns
Pure error	3.4700	3	1.15		
Core total	85.6579	18			

**Table 5:** Structure and ^1^H and ^13^C chemical shift assignments of N-(1-carboxy-ethyl)-phthalamic acid based on the H-H COSY (—) and HMBC (H⟶C) correlations. 
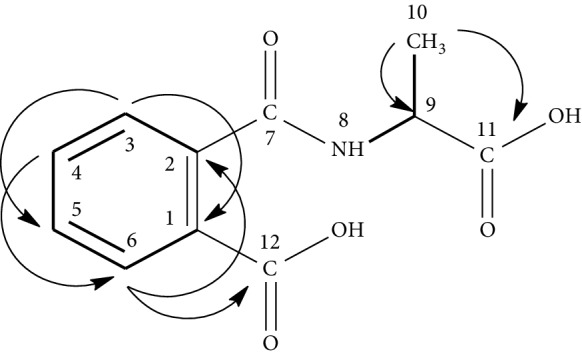

Atom	*δ*C (ppm)	*δ*H (ppm); *m*; *J* (Hz)	H-H COSY	HMBC
1	1218	—	—	—
2	138.0	—	—	—
3	122.4	8.30 (*d*, 8.2)	7.47	123.9
4	132.4	7.47 (*t*, 8.4)	8.30–7.14	126.4–138.0
5	123.9	7.14 (*t*, 7.5)	7.47–7.55	122.4
6	126.4	7.55 (*d*, 7.8)	7.14	132.4–138.0–167.9
7	171.2	—	—	—
8 (NH)	—	6.94 (*d*, 7.8)	4.98	—
9	50.6	4.98 (*m*)	6.94–1.60	19.1–170.5
10	19.1	1.60 (*d*, 6.8)	4.98	50.6–170.5
11	170.5	—	—	—
12	167.9	—	—	—

## Data Availability

The table and figure data used to support the findings of this study are available from the corresponding author upon request (fdonyez@yahoo.com) or can and can be found at https://www.researchgate.net/profile/Donyez_Frikha-Dammak. The complimentary data used to explain support the findings of this study are included within the supplementary information files.

## Supplementary Materials

Supplementary Materials Table S1: composition of fermentation media and antibacterial activity of *P. halophilum* crude extract against *S. enterica*. The experiments were repeated three times for each assay. Table S2: validation of the model using checkpoints. Table S3: GC/MS data of TLC separated F2 fraction. Table S4: NMR data related to the fatty acid compounds in F2. Fig. S1: ^1^H NMR (a) and ^13^C NMR and DEPT (b) spectrum of F2 in CDCl 3 at 400 and 100 MHz, respectively. Representative NMR spectra are from two independent fermentations and isolations. Chemical shifts of residual trifluoroacetic acid are indicated by X. Fig. S2: H-H COSY (a) and expansions of ■HSQC and ■HMBC spectra of F2 in CDCl 3 at 400 MHz (high-field region (b)) and 100 MHz (low-field region (c)) from 2 independent fermentations and isolations.
