# Supplementary material for: Enhancement of Antibacterial Activity of Paludifilum halophilum and Identification of N-(1-Carboxy-ethyl)-phthalamic Acid as the Main Bioactive Compound
Source: Biomed Res Int. 2020 Feb 12;2020:4805706. doi: 10.1155/2020/4805706 (PMC7038168; doi:10.1155/2020/4805706)
Supplement: Supplementary Materials — Table S1: composition of fermentation media and antibacterial activity of P. halophilum crude extract against S. enterica. The experiments were repeated three times for each assay. Table S2: validation of the model using checkpoints. Table S3: GC/MS data of TLC separated F2 fraction. Table S4: NMR data related to the fatty acid compounds in F2. Fig. S1: 1H NMR (a) and 13C NMR and DEPT (b) spectrum of F2 in CDCl 3 at 400 and 100 MHz, respectively. Representative NMR spectra are from two independent fermentations and isolations. Chemical shifts of residual trifluoroacetic acid are indicated by X. Fig. S2: H-H COSY (a) and expansions of ■HSQC and ■HMBC spectra of F2 in CDCl 3 at 400 MHz (high-field region (b)) and 100 MHz (low-field region (c)) from 2 independent fermentations and isolations. [file 4805706.f1.docx]

**Supplementary material**

**Enhancement of antibacterial activity of *Paludifilum halophilum* and identification of N-(1-carboxy-ethyl)-phthalamic acid as the main bioactive compound**

Donyez Frikha-Dammak · Jawhar Fakhfakh · Dalel Belhaj · Emna Bouattour · Houda Ayadi · Moncef Chaabouni · Habib Ayadi · Sami Maalej

**D. Frikha-Dammak · (🖂) D. Belhaj · H. Ayadi · H. Ayadi · S. Maalej** Laboratory of Marine Biodiversity and Environment (LR18ES/30), BP 1171, CP 3000, Sfax, Tunisia **J. Fakhfakh** University of Sfax, Laboratory of Organic Chemistry (LR17ES08) Natural Substances Section, BP 1171, CP 3000, Sfax, Tunisia

**M. Chaabouni**  University of Sfax, Engineering Laboratory of Environment and Ecotechnology (LR16ES19), BP 1173, CP 3038, Sfax, Tunisia

**Corresponding author :**

**Affiliation** : University of Sfax, Laboratory of Marine Biodiversity and Environment (LR18ES/30), BP 1171, CP 3000, Sfax, Tunisia

**e-mail address** : fdonyez@yahoo.com

**Telephone number : +**216 44999994

**Fax number :** 216 74 274437

**Table S1** List**,** composition of fermentation media and antibacterial activity of *P. halophilum* crude extract against *S. enterica.* The experiments were repeated three times for each assay

| Fermentation  medium | Composition (g/L) | Antibacterial activity (mean ± SD, mm) |
| --- | --- | --- |
| **Bennett’s** | Glucose (10g), Meat extract (1 g), Yeast extract (1 g), Peptone (2 g), NaCl (100 g), Agar (20 g) | 9.0 ± 0.62 |
| **GYP** | Glucose (10 g), Yeast extract (10 g), Peptone (5 g), CaCO_3_ (3 g), K_2_HPO_4_ (0.5 g), MgSO_4_,7H_2_O (0.5g),  NaCl (100 g), Agar (20 g) | 7.33 ± 0.57 |
| **GYM** | Glucose (4 g), Yeast extract (4 g), Malt extract (10 g), CaCO_3_ (2 g), NaCl (100 g), Agar (20 g) | 5.33 ± 0.57 |
| **NPB** | Glucose (4 g), Yeast extract (4 g), Malt extract (2 g), CaCO_3_ (0.5 g), Agar (20 g), NaCl (100 g) | 6.0 ± 0.57 |
| **GSA** | Glycerol (20 g), Starch (20 g), Peptone (10 g), Meat extract (5 g), CaCO_3_ (3 g), NaCl (100 g), Agar (20 g) | 7.66 ± 0.57 |

Composition of five different media named Bennett’s agar (Bennett’s), Glucose Yeast Peptone Agar (GYP), Glucose Yeast Malt agar (GYM), NPB agar (NPB) and Glycerol Starch Agar (GSA) were tested for the biomass and the antibacterial activity.

**Table S2:** Validation of the model using check points

| Responses | Y | check Point (17) | check Point (18) | check Point (19) | |
| --- | --- | --- | --- | --- | --- |
| Biomass production (mg/ml) | Y measured | 6.80 | 6.60 | 6.40 |  |
|  | Y predicted | 6.12 | 5.48 | 5.43 |  |
| Antibacterial activity (mm) | Y measured | 12.00 | 11.70 | 12.30 |  |
|  | Y predicted | 11.79 | 11.20 | 12.43 |  |

Table S2 represent three additional independent experiments carried out at three check points. The measured responses are in close agreement with the predicted values

**Table S3**: GC/MS data of TLC separated F2 fraction.

| **N°** | **Retention time** | **Compound** | **%** |
| --- | --- | --- | --- |
| 1 | 12.183 | Heptanedioic acid | 0.98 |
| 2 | 13.26 | Octanedioic acid | 3.14 |
| 3 | 14.323 | Azelaic acid | 6.96 |
| 4 | 15.318 | Sebacic acid | 0.94 |
| 5 | 16.772 | Hexadecanoic acid | 16.75 |
| 6 | 17.20 | 9-Octadecenoic acid | 1.33 |
| 7 | 17.41 | Heptadecanoic acid | 0.93 |
| 8 | 18.324 | Oleic acid | 10.2 |
| 9 | 18.550 | Octadecanoic acid | 10.99 |
| 10 | 20.15 | 11-Eicosenoic acid | 3.53 |

The analysis by GCMS was performed after silylation and revealed the presence of 10 compounds mainly fatty acids. Their identification was based on the comparison of their mass spectra with those from a data library with which they exhibited more than 90 % similarity.

**Table S4:**  NMR data related to the fatty acid compounds in F2

|  | **δH (ppm); *mult* ; J (Hz)** | **δC (ppm)** | **H-H COSY** | **HMBC** |
| --- | --- | --- | --- | --- |
| Methyl groups | 0.89 (*t*, 6.6 ) | 14.1 | 1.29 | 22.5 – 31.7 |
| Methylenes, except the following | 1.29 (*m*) | 29.0 – 29.1 - 29.2 - 29.3 - 29.4 - 29.5 - 29.6 – 29.7 | 0.89 – 1.60 | 24.7 – 24.9 – 34.1 |
| Methylenes in α to the methyl groups | 1.29 (*m*) | 22.5 | *^a^* | 14.1 – (29.0 to 29.7) - 31.7 |
| Methylenes in β to the methyl groups | 1.29 (*m*) | 31.7 | *^a^* | 14.1 – 22.5 - (29.0 to 29.7) |
| Methylenes in α to the carbonyl groups | 2.37 (*m*) | 34.1 | 1.60 | 24.7 – 24.9 - (29.0 to 29.7) –175.1 |
| Methylenes in β to the carbonyl groups | 1.63 (*m*) | 24.7 – 24.9 | 1.29 – 2.34 | (29.0 to 29.7) –175.1 |
| C=O | - | 175.1 | - | - |

Table S4 show the connections between the multiplet methyle group methylenes; the Methylenes in α and β to the methyl groups and the methylene groups in α and β position to the carboxyl groups in **δH and δC**, as well as the study of H-HCOSY and the HSQC confirm the global fatty acids’ skeletons.

**Fig. S1:** ^1^H NMR (a) and ^13^C NMR and DEPT (b) spectrum of F2 in CDCl_3_ at 400 and 100 MHz, respectively. Representative NMR spectra are from two independent fermentations and isolations. Chemical shifts of residual trifluoroacetic acid are indicated by an X.

The 1H NMR spectrum in figure S1a show a renowned intense signals related, mainly, to saturate fatty acids, when the figure S1 b illustrate the related resonances on the 13C NMR spectrum who show the raise of the carbonyl groups of the acid moieties.

**Fig. S2** H-H COSY (a) and expansions of ■HSQC and ■HMBC spectra of F2 in CDCl_3_ at 400MHz (high field region (b)) and (low field region (c) from 2 independent fermentations and isolations

The H-H COSY spectrum rises connections between the multiplet (CH3) and the intense signal (CH2), the HSQC spectrum explain makes correlation with the doublet of three protons and the HMBC represent the connectivity between the multiplex (C-H).
